# Serum-Derived Extracellular Vesicles Protect Against Acute Myocardial Infarction by Regulating miR-21/PDCD4 Signaling Pathway

**DOI:** 10.3389/fphys.2018.00348

**Published:** 2018-04-05

**Authors:** Huanyu Gu, Zhuyuan Liu, Yongqin Li, Yuan Xie, Jianhua Yao, Yujiao Zhu, Jiahong Xu, Qiying Dai, Chongjun Zhong, Hao Zhu, Shengguang Ding, Lei Zhou

**Affiliations:** ^1^Department of Cardiology, The First Affiliated Hospital of Nanjing Medical University, Nanjing, China; ^2^Cardiac Regeneration and Ageing Lab, School of Life Science, Shanghai University, Shanghai, China; ^3^Department of Cardiology, Shanghai Tongji Hospital, Tongji University School of Medicine, Shanghai, China; ^4^Department of Cardiology, Shanghai Tenth Hospital, Tongji University School of Medicine, Shanghai, China; ^5^Department of Thoracic and Cardiovascular Surgery, The Second Affiliated Hospital of NanTong University, Nantong, China

**Keywords:** extracellular vesicles, miR-21, acute myocardial infarction, PDCD4, cardiomyocytes apoptosis

## Abstract

Acute myocardial infarction (AMI) represents a leading cause of morbidity and mortality worldwide. Extracellular vesicles (EVs) are being recognized as a promising therapeutic approach in protecting against MI. Serum is a rich source of EVs, which transports various microRNAs (miRNAs, miRs). EVs from serum have been shown beneficial for protecting against ischemia-reperfusion injury; however, their roles in AMI are unclear. In addition, whether a miRNA might be responsible for the effects of serum EVs on protecting against AMI is undetermined. Here, we demonstrated that serum EVs significantly reduced cardiomyocytes apoptosis in both cellular and mouse models of AMI, and dramatically attenuated the infarct size in mouse hearts after AMI. Inhibition of miR-21 was shown to reduce the protective effects of serum EVs in inhibiting cardiomyocytes apoptosis. miR-21 was decreased in mouse hearts after AMI, while serum EVs increased that. In addition, the programmed cell death 4 (PDCD4) expression was identified as a target gene of miR-21. Therefore, our study showed the protective effects of serum EVs on AMI, and provided a novel strategy for AMI therapy.

## Introduction

Acute myocardial infarction (AMI) is one of the leading causes of morbidity and mortality worldwide, which results from coronary artery occlusion and characterized by plaque rupture-induced thrombosis and subsequent hypoxic ischemic injury (Benjamin et al., 2017). Cardiomyocytes apoptosis occurs frequently during the pathological process of AMI, leading to cardiomyocytes loss (Palojoki et al., 2001; Nabel and Braunwald, 2012). Therefore, approaches that inhibit cardiomyocytes apoptosis are promising therapeutic approach for ischemic heart injury and AMI.

Extracellular vesicles (EVs) are being recognized as new candidates with emerging roles in promoting cardiac repair during AMI (Boulanger et al., 2017). EVs are extracellular membrane-bound vesicles (~40–100 nm in diameter), secreted by most cell types (Simons and Raposo, 2009; Mathivanan et al., 2010). EVs contain RNAs, proteins, and lipids, some of which are specific due to its cellular origin, whereas others are ubiquitous to all EVs such as tetraspannins (CD9, CD63, CD81) (Kowal et al., 2016) and heat shock proteins (HSC70) (Vlassov et al., 2012). EVs can mediate communication between cells, tissues, and organs, inducing physiological and pathological changes (Valadi et al., 2007; Skog et al., 2008). The important functions of EVs include, but not limited to, regulating cardiomyocytes survival, cardiomyocytes communication with fibroblasts and endothelial cells, angiogenesis and cardiac remodeling in heart tissues (Cosme et al., 2013; Gaceb et al., 2014). Mesenchymal stem cell-derived EVs reduce cardiomyocytes loss after myocardial ischemia/reperfusion injury via activating PI3K/AKT pathway (Arslan et al., 2013). EVs from human cardiac progenitor cells exhibited less cardiomyocytes apoptosis and improved cardiac function after AMI (Barile et al., 2014). EVs from cardiosphere-derived cells reduced scarring and attenuated adverse remodeling after AMI and chronic MI in porcine (Gallet et al., 2017). These studies suggested the important roles of EVs in cardiac protection against ischemic cardiovascular diseases. Therefore, EVs could serve as new therapeutic approach for AMI. To date, most studies mainly focused on the function of exogenous EVs derived from *in vitro* cultured-pluripotent cells. Recently, EVs from serum have been shown to prevent the myocardium from ischemia-reperfusion injury (Vicencio et al., 2015). However, limited knowledge on the roles of endogenous EVs in cardiac protection during AMI is available.

More importantly, EVs are abundant in blood serum, serving as a rich source of circulating microRNAs (miRNAs, miRs) (Kalra et al., 2013; Moldovan et al., 2013). miRNAs are a class of small non-coding RNAs that post-transcriptionally down-regulate gene expression (Jing et al., 2005) and play important roles in cardiovascular diseases (Ong et al., 2018). miRNAs in EVs have been shown to regulate important processes of AMI. EVs from mesenchymal stem cells (MSCs) following ischemic preconditioning were abundant with miR-22 and reduced cardiomyocytes apoptosis and cardiac fibrosis after AMI (Feng et al., 2014). miR-294 enriched in embryonic stem cell-EVs stimulated and enhanced the proliferation of cardiac progenitor cells and cardiomyocytes after AMI (Khan et al., 2015). EVs from cardiac progenitor cells dramatically impart cardiac protection in AMI and the underlying mechanism may involve miR-210 and miR-132 (Barile et al., 2014). miR-21 was significantly decreased after AMI in heart tissue and miR-21 agomiRs reduced myocardial infarct size and cardiomyocytes apoptosis during chronic MI (Gu et al., 2015) and AMI (Huang et al., 2016). miR-21 in cardiac progenitor cell-derived EVs had crucial roles in preventing oxidative stress-related cardiomyocytes apoptosis *in vitro* (Xiao et al., 2016). Besides, EVs derived from different ways vary in their contents and function, and even EVs of the same origin might have reversed effects in different pathological conditions. Hence, it is still undetermined that whether endogenous EVs from serum have protection roles in AMI and whether miR-21 is responsible for the protection effects.

Here, we investigated the protective effects of the serum EVs in both cellular and mouse models of AMI, which was mainly through shuttling miR-21 and inhibited programmed cell death 4 (PDCD4) expression in cardiomyocytes. Our study may provide a new potential strategy for AMI therapy.

## Methods and materials

### Safety

For research involving biohazards, biological select agents, toxins, restricted materials or reagents, we have carried out the standard biosecurity and institutional safety procedures over the course of our study.

### Animals

All 8-week-old male C57BL/6 mice used in this study were purchased from Shanghai Laboratory Animal Center and maintained at the Experimental Animal Center of Shanghai University (Shanghai, China) with specific pathogen-free (SPF) condition. Protocols of this study were all approved by ethical committees of Nanjing Medical School and all animal experiments were in accordance with the guidelines on the use and care of laboratory animals for biomedical research which was published by National Institutes of Health (No.85-23, revised 1996).

### Intra-myocardial injection of EVs and mouse model of acute myocardial infarction

Myocardial injection of serum EVs was performed before mice were subjected to acute myocardial infarction (AMI) surgery. Briefly, 10 μg EVs diluted in 25 μl PBS isolated from wild-type mice were injected in the left ventricle free wall before AMI surgery. Control mice were treated with intra-myocardial injection of 25 μl PBS. Then the mice were conducted under anesthesia with pentobarbital sodium, and the left anterior descending coronary artery (LAD) was ligated with 7-0 silk thread, sham group was generated by the same process without LAD ligation. The mice were sacrificed at 24 h after the ligation and the heart samples were collected according to infarct area and border area.

### Serum collection

Protocols of all human investigations used in this study were reviewed and approved by the Ethics Committee of Nanjing Medical University. Written informed consent was obtained from the participants in this human population study which conformed to the principles outlined in the Declaration of Helsinki. 5 ml samples of human blood were obtained by percutaneous cubital venipuncture drawn in silicone-coated serum tubes with increased silica act clot activator. One milliliter samples of mouse blood were collected from orbit. The blood samples were centrifuged at 3,000 g for 15 min at 4°C. After discarding the lipid-rich topmost layer, the serum was carefully transferred to new centrifuge tubes.

### EV purification

EVs were extracted from serum using SBI ExoQuick kit (GENETIMES, Shanghai, China). Briefly, 250 ul serum samples from mice or human blood were mixed with 63 μl ExoQuick reagent for 30 min and centrifuged at 1,500 g for 30 min at 4°C to remove the supernatant. At last, resuspend the pellets in 100 μl PBS and stored at −80°C.

### TTC staining

Inject 1 ml Evans blue (BioSharp, Anhui, China) into inferior vena and remove the heart. Then the heart was cut into six slices and stained with 1% triphenyltetrazolium chloride (TTC, Amresco, OHIO, USA) at 37°C for 15 min.

### Infarct size determination

The myocardial infarct size was quantified with ratio of area-at-risk relative to area of left ventricle (AAR/LV) and ratio of infarct size relative to AAR(INF/AAR), the size of area was calculated by Image J Software.

### Cell culture and treatment

Primary neonatal rat cardiac myocytes (NRCMs) were isolated and cultured as previously described (Liu et al., 2015). NRCMs were treated with 10 μg/ml human's or mouse's EVs in serum-free DMEM for 24 h. Cardiomyocytes were transfected with miR-21 mimics and inhibitors for 48 h, and/or with siRNAs for PDCD4 (sequence: 5'-CACTGACCCTGACAATTTAAG-3′), using Lipofectamine™ 2000 (Invitrogen, MA, USA) according to the manufacturer's instruction. miR-21 mimics (50 nM), miR-21 inhibitors (100 nM) and their negative controls were synthesized by Ribobio Co. (Guangzhou, China).

### Oxygen-glucose deprivation and reperfusion (OGD/R) model

NRCMs were deprived oxygen and glucose for 8 h, followed by 12 h recovery. To achieve OGD, NRCMs were cultured in glucose-free DMEM (Gibco) and an air-tight chamber with a humidified hypoxic atmosphere which contained 5% CO_2_ and 95% N_2_ at 37°C. After 8 h of exposure to hypoxia, NRCMs were transferred to a normal incubator with glucose-containing DMEM medium for recovery.

### TUNEL assay

Terminal deoxyribonucleotidyl transferase-mediated dUTP *in situ* nick-end labeling (TUNEL) assay was used to detect NRCM's apoptotic nuclei. *In situ* Cell Death Detection Kit (Vazyme, Nanjing, China) was used according to kit's instruction.

### Immunofluorescent staining

Cells were fixed in 4% paraformaldehyde (PFA) followed by washing with PBS. After being blocked with 5% BSA, cells were incubated with primary antibody (α-actinin, 1:200; Cat. A7811, Sigma) at 4°C overnight. After being washed with PBS, cells were incubated with secondary antibody (CY3, 1:200; Cat. 715-165-151, JACKSON) for 2 h at room temperature, followed by DAPI (1:2,000; KeyGEN Biotech, Nanjing, China) staining. All images were taken by fluorescence microscope under a magnification of 200×.

### Western blot

Heart or cell samples were lysed in RIPA buffer (KeyGEN, Nanjing, China) with protease inhibitor cocktail (KeyGEN) and concentration of protein sample was quantified by BCA Protein Assay Kit (TaKaRa). Thirty micrograms of total protein was separated by 10% SDS-polyacrylamide gel electrophoresis gels, then proteins were transferred to polyvinylidene difluoride membranes. After being blocked with 5% milk for 1 h at room temperature, the membranes were incubated with primary antibodies overnight at 4°C, and the appropriate horseradish peroxidase-conjugated secondary antibody was followed. After all, all protein bands were detected by BioRad luminescent imaging system with ECL Chemiluminescent Kit (Thermo Fisher). The primary antibodies used in this study are as follows: anti-Bax (1:1,000; Cat. A0207, ABclonal, Wuhan, China), anti-Bcl-2 (1:1,000; Cat. A0208, ABclonal), anti-Caspase3 (1:1,000; Cat. 9662, Cell Signaling Technology, Boston, MA), anti-CD63 (1:1,000; Cat. A5271, ABclonal), anti-PDCD4 (1:1,000; Cat. AB38428, Absci) and anti-β-actin (1:1,000; Cat. AC026, ABclonal).

### Quantitative reverse transcription polymerase chain reactions (qRT-PCRs)

Total RNAs of cells and heart tissues were conducted by RNeasy Mini Kit (Qiagen, Hilden, Germany) according to manufacturer's instruction. To analyze mRNA expression levels of PDCD4, cDNA was synthesized using Takara PrimeScript 1st Strand cDNA Synthesis Kit and subjected to 40 cycles of quantitative PCR with Takara SYBR Premix Ex TaqTM (TliRNaseH Plus, Japan). 18S was used as an internal control. To determine the miR-21 expression levels, Bulge-Loop miRNA qPCR Primer Set (RiboBio) was used with Takara SYBR Premix Ex Taq (TliRNaseH Plus) in a BioRad CFX96 Real-Time PCR Detection System. 5S was used as an internal control. Primer sequences (forward and reverse) used in this study are as follows:

rno-PDCD4, 5′-TGAGCACGGAGATACGAACGA-3′

and 5′-GCTAAGGACACTGCCAACACG-3′;

rno-18S, 5′-TCAAGAACGAAAGTCGGAGG-3′

and 5′-GGACATCTAAGGGCATCAC-3′;

mmu-miR-21a-5p, 5′-CGCGCGCGTAGCTTATCAGACTGA-3′

and 5′-ATCCAGTGCAGGGTCCGAGG-3′;

mmu-5S, 5′-GTCTACGGCCATACCACCCTGAACG

and ATCCAGTGCAGGGTCCGAGG.

The relative expression levels of were calculated in 2^−ΔΔCt^ method.

### Statistical analysis

All data were shown as mean ± standard error of mean (SEM). Paired groups were compared by independent-samples *t*-test, and one-way analysis of variance (ANOVA) test was used to compare among three or four groups, followed by Bonferroni's *post-hoc* test. All analyses were performed using statistical software SPSS 19.0 (IBM Armonk, New York, USA). *P* < 0.05 was considered to be statistically significant.

## Results

### Serum EVs protects against cardiomyocytes apoptosis induced by oxygen-glucose deprivation and reperfusion

**Figure 1:**
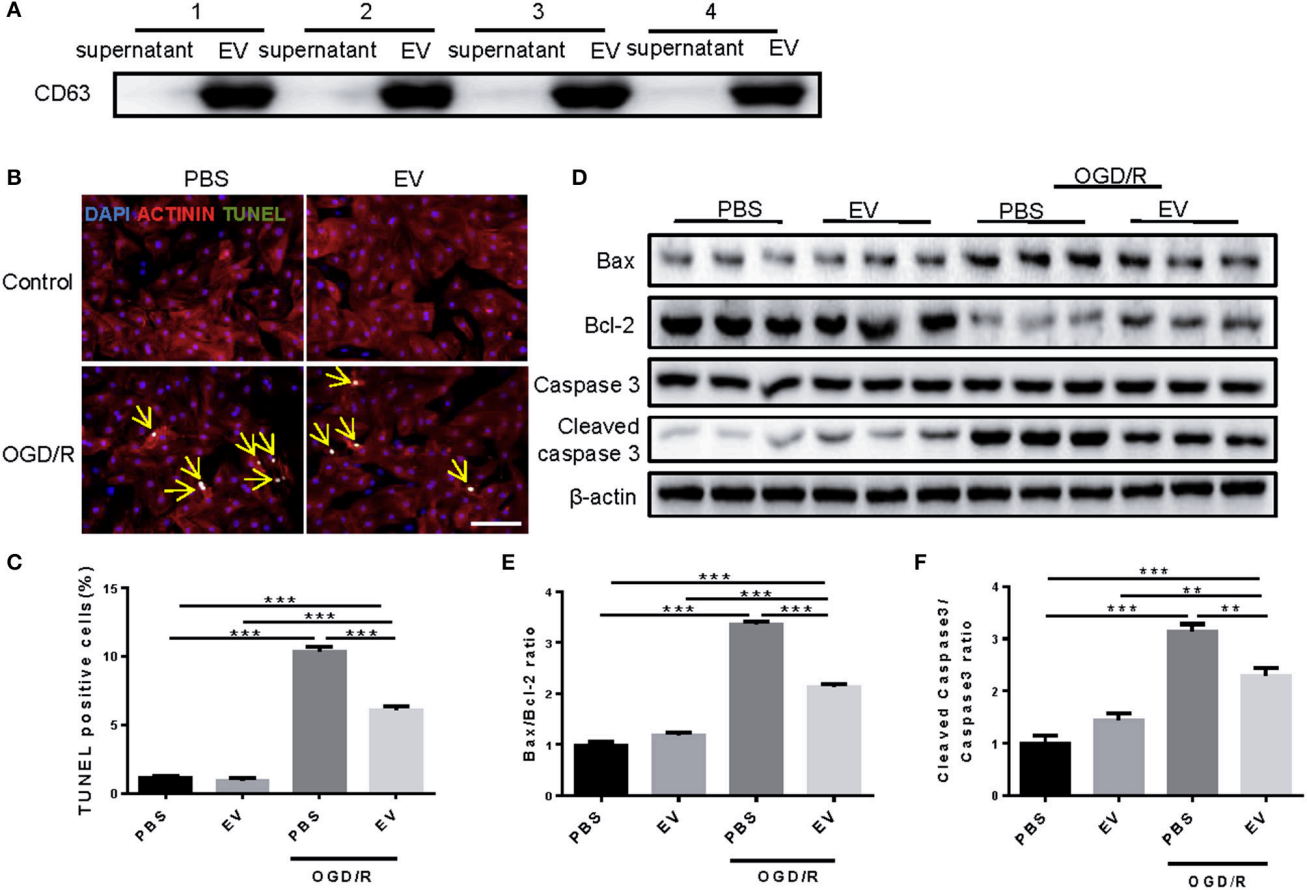
Human serum extracellular vesicles inhibit OGD/R induced apoptosis in NRCMs. **(A)** Western blot for CD63 in supernatant and EVs of human serum (*n* = 4). **(B)** Terminal deoxynucleotidyl transferase-mediated nick end labeling (TUNEL) assay for apoptosis in NRCMs treated with OGD/R (*n* = 4). Scale bar: 100 μm. **(C)** Analysis for percentage of TUNEL positive cells (*n* = 4). **(D–F)** Western blot for Bax, Bcl-2, Caspase 3, and Cleaved caspase 3, and quantitative analysis for Bax/Bcl-2 and Cleaved caspase 3/Caspase 3 ratio. β-actin was used as a loading control (*n* = 6). **P < 0.01; ***P < 0.001. EVs, extracellular vesicles.

**Figure 2:**
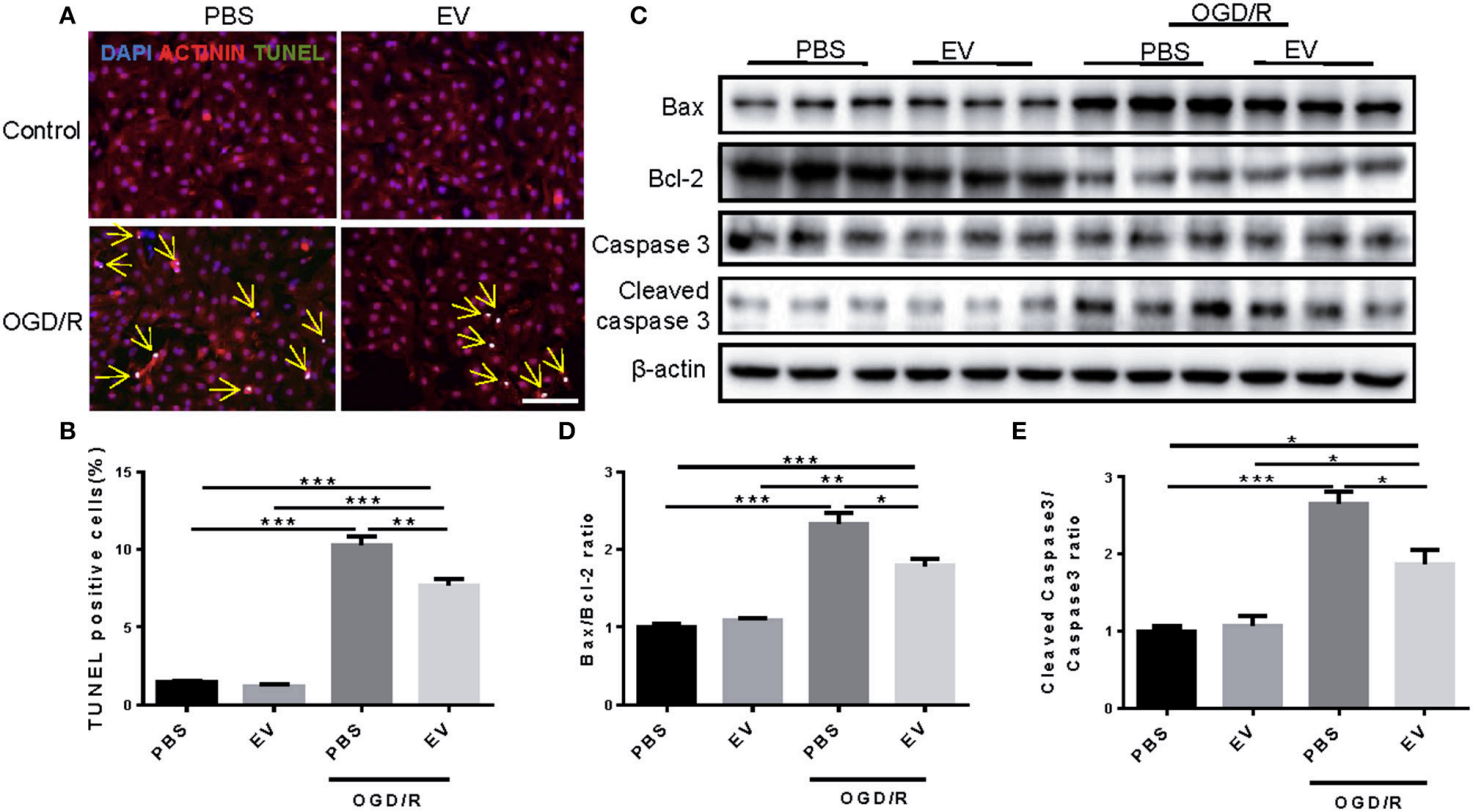
Mouse serum extracellular vesicles inhibit OGD/R induced apoptosis in NRCMs. **(A,B)** Terminal deoxynucleotidyl transferase-mediated nick end labeling (TUNEL) staining of NRCMs treated with EVs extracted from mouse serum under the condition of OGD/R (*n* = 4). Scale bar: 100 μm. **(C–E)** Western blot for Bax, Bcl-2, Caspase 3, and Cleaved caspase 3, and quantitative analysis for Bax/Bcl-2 and Cleaved caspase 3/Caspase 3 ratio. β-actin was used as a loading control (*n* = 6). ^*^0.01 < *P* < 0.05; ^**^0.001 < *P* < 0.01; ^***^P < 0.001. EVs, extracellular vesicles.

EVs were purified from the blood of four healthy human volunteers using a standard protocol. Western blot confirmed the highly expression of the tetraspanin protein CD63 in EV-rich fractions, which is a marker protein for EVs in human samples (Figure 1A). To investigate whether serum EVs can protect cardiomyocytes from apoptosis, we used oxygen-glucose deprivation and reperfusion (OGD/R) model in primary neonatal rat cardiomyocytes (NRCMs). After treated with OGD/R, the NRCMs were harvested for terminal deoxynucleotidyl transferase nick-end labeling (TUNEL) staining and western blot analysis. TUNEL staining showed that human serum EVs reduced the number of TUNEL-positive cells in NRCMs challenged with OGD/R (Figures 1B,C), with deceased Bax/Bcl-2 and cleaved caspase-3/caspase-3 ratio as determined by western blot analysis (Figures 1D–F). Similar results were obtained using EVs from mouse serum (Figure 2). These results suggested that human and mouse serum EVs could attenuate cardiomyocytes apoptosis induced by OGD/R.

### miR-21 in the human serum-derived EVs is involved in preventing cardiomyocytes from OGD/R-induced apoptosis

**Figure 3:**
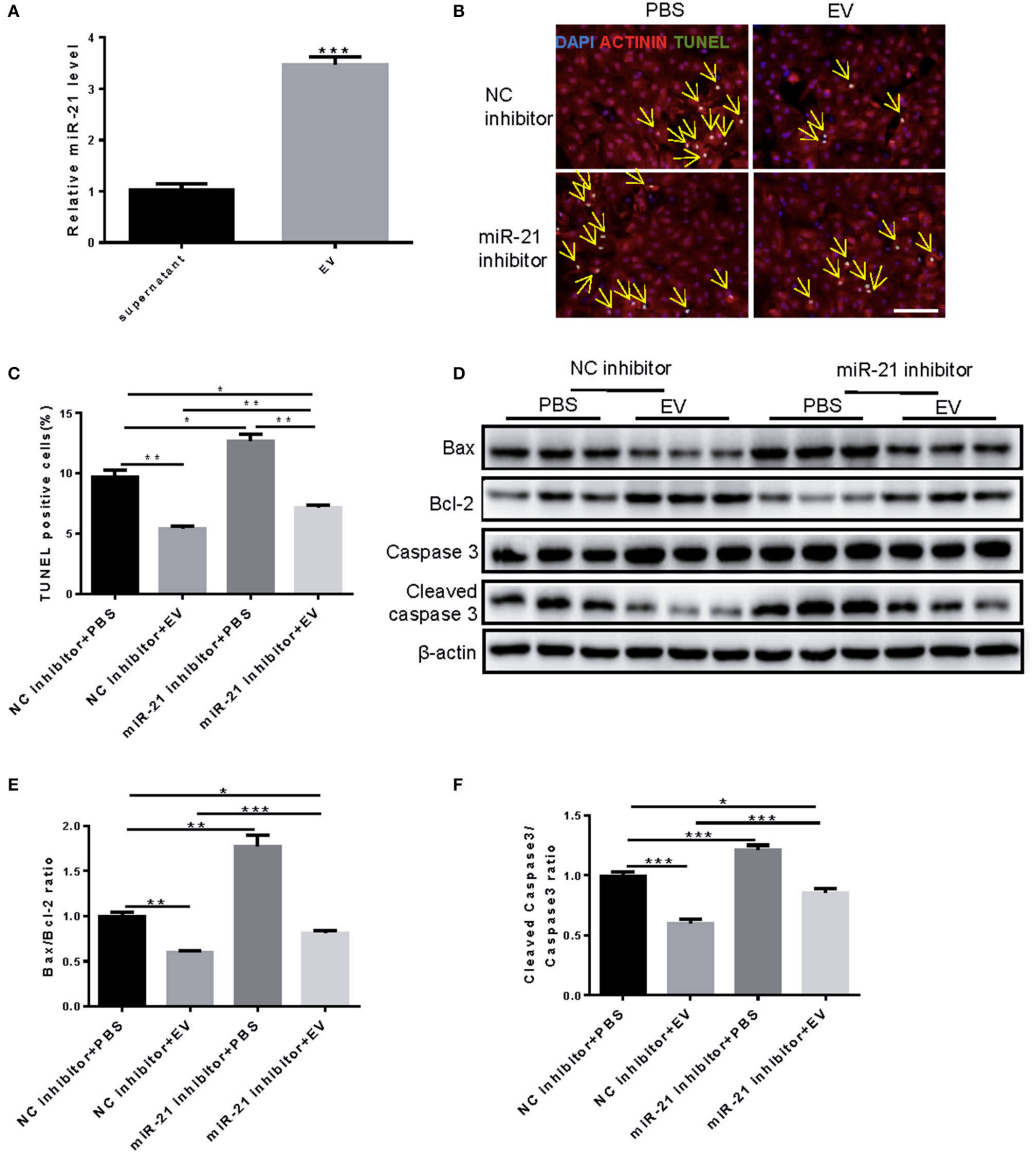
Knockdown of miR-21 partially rescue the inhibition of OGD/R-induced NRCM apoptosis by human serum extracellular vesicles. **(A)** qRT-PCRs for expression of miR-21 in supernatant and EVs extracted from mouse serum. *n* = 4. **(B)** Terminal deoxynucleotidyl transferase-mediated nick end labeling (TUNEL) assay for apoptosis in NRCMs (*n* = 4). Scale bar: 100 μm. **(C)** Analysis for percentage of TUNEL positive cells (*n* = 4). **(D–F)** Western blot for Bax, Bcl-2, Caspase 3, and Cleaved caspase 3, and quantitative analysis for Bax/Bcl-2 and Cleaved caspase 3/Caspase 3 ratio. β-actin was used as a loading control (*n* = 6). *P < 0.05; **P < 0.01; ***P < 0.001. EVs, extracellular vesicles.

miR-21 from cardiac progenitor cell-secreted EVs have been shown important for reducing H_2_O_2_-induced cell apoptosis in H9C2 cells (Xiao et al., 2016). And our result showed that miR-21 was highly enriched in serum-derived EVs compared with the supernatant (Figure 3A). This provided us a miRNA candidate that might be responsible for the anti-apoptosis roles of serum EVs in NRCMs under OGD/R condition. To detect whether miR-21 was involved in the effects of EVs on OGD/R-induced apoptosis of cardiomyocytes, TUNEL assay (Figures 3B,C) and western blot (Figures 3D–F) were performed after treatment with miR-21 inhibitors and/or human serum EVs in NRCMs under OGD/R condition. The results demonstrated that human serum EVs obviously decreased TUNEL-positive NRCMs, Bax/Bcl-2, and cleaved caspase-3/caspase-3 ratio, whereas miR-21 inhibitors attenuated the reduction of TUNEL-positive NRCMs as well as Bax/Bcl-2 and cleaved caspase-3/caspase-3 ratio induced by serum EVs (Figure 3). Therefore, miR-21 is at least partly responsible for the protective effects of human serum EVs in apoptosis of NRCMs induced by OGD/R.

### Inhibition of PDCD4 mediates the anti-apoptotic effects of miR-21 and human serum EVs in cardiomyocytes under OGD/R

**Figure 4:**
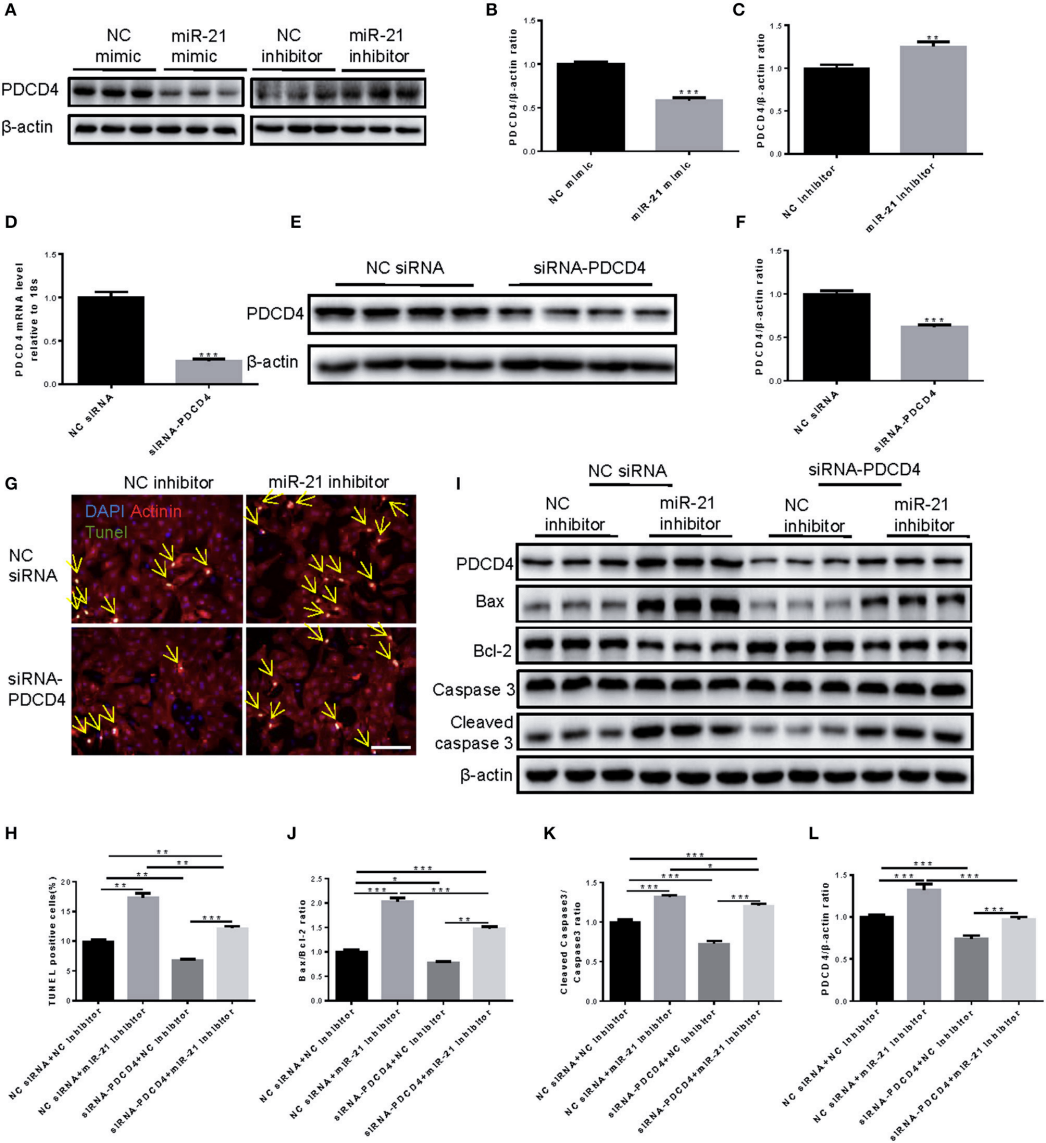
PDCD4 is a target gene of miR-21 in OGDR-induced NRCM apoptosis. **(A–C)** Western blot and quantitative analysis for PDCD4 after miR-21 mimic and miR-21 inhibitor treatments in NRCMs (*n* = 6). **(D)** mRNA expression levels of PDCD4 was decreased in NRCMs after siRNA-PDCD4 treatment (*n* = 6). **(E,F)** Western blot and quantitative analysis for PDCD4 after siRNA-PDCD4 treatments in NRCMs (*n* = 4). **(G)** Terminal deoxynucleotidyl transferase-mediated nick end labeling (TUNEL) assay for apoptosis in NRCMs (*n* = 4). Scale bar: 100 μm. **(H)** Analysis for percentage of TUNEL positive cells (*n* = 4). **(I–L)** Western blot for PDCD4, Bax, Bcl-2, Caspase 3, and Cleaved caspase 3, and quantitative analysis for PDCD4, Bax/Bcl-2, and Cleaved caspase 3/Caspase 3 ratio. β-actin was used as a loading control (*n* = 6). ^*^0.01 < *P* < 0.05; ^**^0.001 < *P* < 0.01; ^***^P < 0.001.

PDCD4 was a target gene of miR-21 in H9C2 cells (Xiao et al., 2016). To confirm whether PDCD4 is a target gene of miR-21 in NRCMs, the expression levels of PDCD4 in NRCMs treated with miR-21 mimics or inhibitors were determined by western blot. The results revealed that miR-21 mimics reduced while miR-21 inhibitors elevated PDCD4 expression (Figures 4A–C). Then we synthesized the specific siRNAs of PDCD4, which dramatically decreased PDCD4 mRNA (Figure 4D) and protein levels in NRCMs (Figures 4E,F). To further address whether miR-21 reduced cell apoptosis through inhibiting the expression of PDCD4, we performed rescue experiments. TUNEL assay (Figures 4G,H) and western blot (Figures 4I–L) suggested that PDCD4 siRNAs could obviously suppress the effects of miR-21 inhibitors in promoting NRCM apoptosis stimulated by OGD/R.

**Figure 5:**
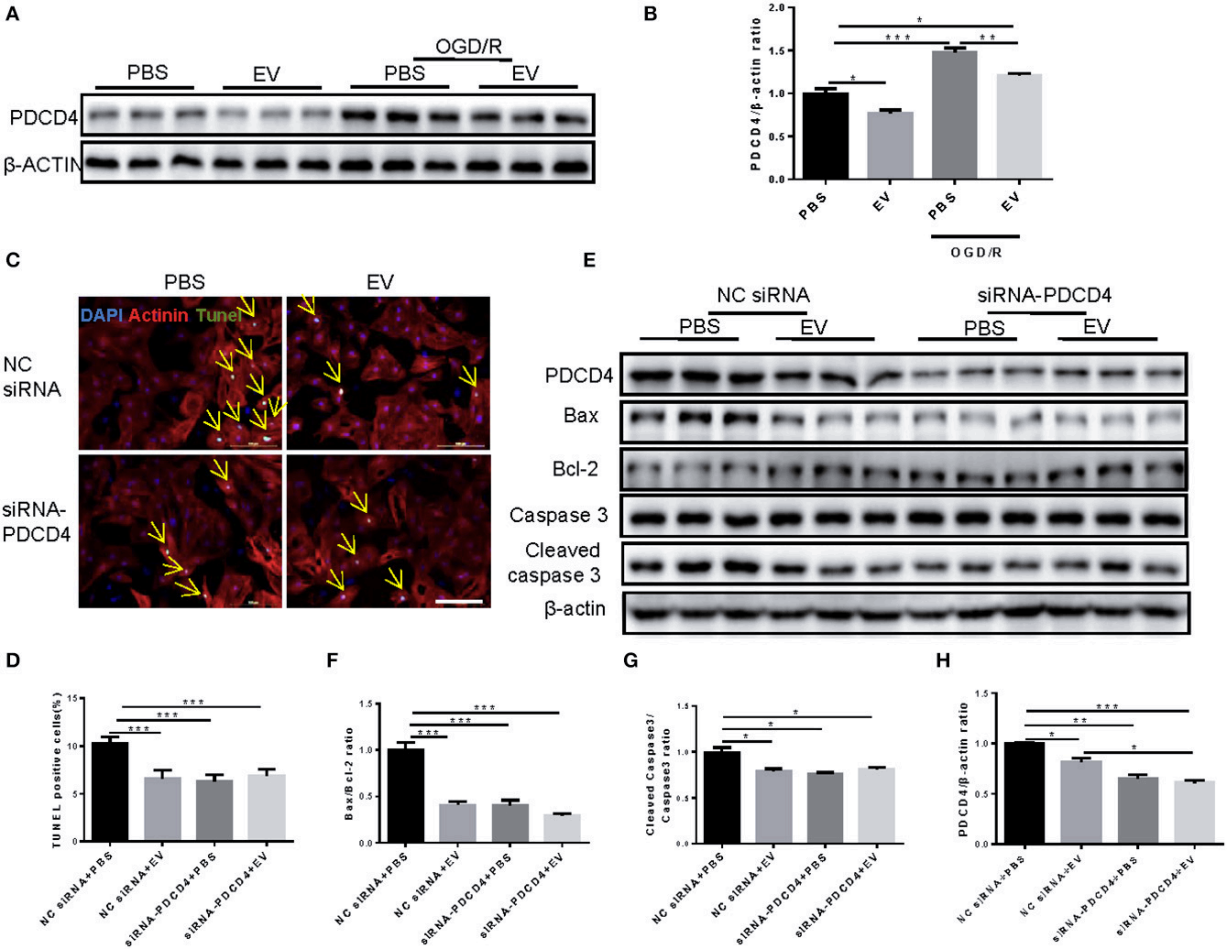
Knockdown of PDCD4 inhibited the protection of human extracellular vesicles against OGDR-induced NRCM apoptosis. **(A,B)** Western blot for PDCD4 and quantitative analysis for PDCD4/β-actin ratio (*n* = 6). **(C)** Terminal deoxynucleotidyl transferase-mediated nick end labeling (TUNEL) assay for apoptosis in NRCMs treated with OGD/R (*n* = 4). Scale bar: 100 μm. **(D)** Analysis for percentage of TUNEL positive cells (*n* = 4). **(E–H)** Western blot for PDCD4, Bax, Bcl-2, Caspase 3 and Cleaved caspase 3, and quantitative analysis for PDCD4, Bax/Bcl-2 and Cleaved caspase 3/Caspase 3 ratio. β-actin was used as a loading control (*n* = 6). ^*^0.01 < *P* < 0.05; ^**^0.001 < *P* < 0.01; ^***^P < 0.001. EVs, extracellular vesicles.

Consistently, human serum EVs showed strong effects on reducing PDCD4 expression both in NRCMs under normal conditions and NRCMs under OGD/R stimulation (Figures 5A,B). However, co-transfection of PDCD4 siRNAs and human serum EVs did not exert an additive effect in reducing OGD/R-induced cardiomyocytes apoptosis as determined by TUNEL assay (Figures 5C,D) and western blot (Figures 5E–H), confirming that inhibition of PDCD4 contributes to the anti-apoptotic effects of miR-21 and serum EVs in cardiomyocytes.

### Serum EVs from mouse elevated miR-21 expression and attenuated the AMI-induced infarct area and cell apoptosis in mice

**Figure 6:**
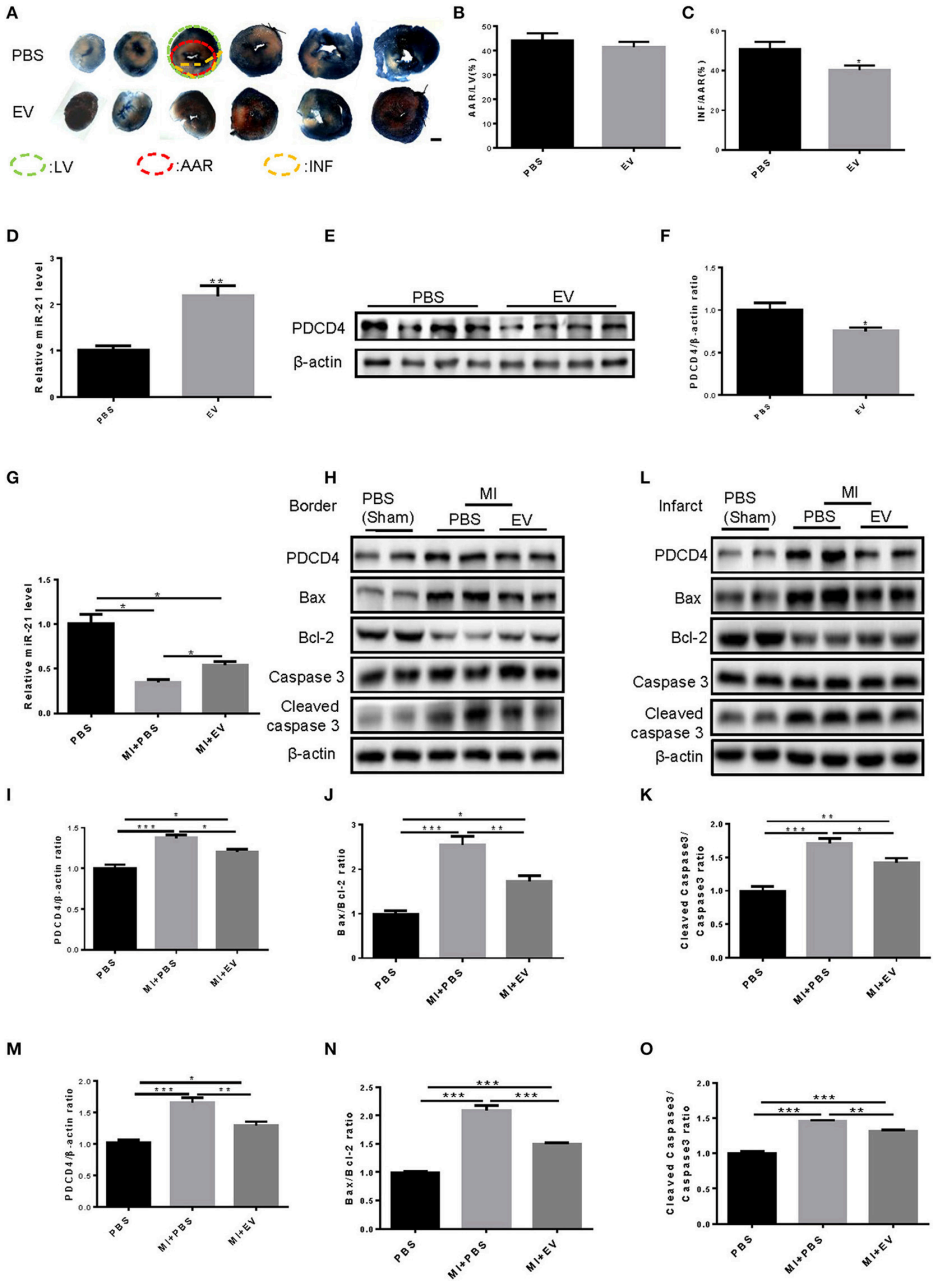
Mouse serum extracellular vesicles increased miR-21 levels, reduced the AMI-induced infarct area and attenuated cardiomyocytes apoptosis in mouse. **(A)** Photographs of TTC staining of six slices from mice cardiac tissues (*n* = 4). Scale bar, 1,000 μm. **(B,C)** Analysis of AAR/LV and INF/AAR ratio to define the infarct area of hearts from mice treated with MI and EVs extracted from mice serum (*n* = 4). **(D)** qRT-PCRs for expression of miR-21 in mouse heart tissues at 24 h after the injection of EVs. **(E,F)** Western Blot for expression of PDCD4 in mouse heart tissues at 24 h after the injection of EVs. *n* = 4; **(G)** Quantitative real time-polymerase chain reactions (qRT-PCRs) for expression of miR-21 (*n* = 4). **(H–K)** Western blot of border tissues for PDCD4, Bax, Bcl-2, Caspase 3, and Cleaved caspase 3, and quantitative analysis for PDCD4, Bax/Bcl-2 and Cleaved caspase 3/Caspase 3 ratio. β-actin was used as a loading control (*n* = 4). **(L–O)** Western blot of infarct tissues for PDCD4, Bax, Bcl-2, Caspase 3 and Cleaved caspase 3, and quantitative analysis for PDCD4, Bax/Bcl-2, and Cleaved caspase 3/Caspase 3 ratio. β-actin was used as a loading control (*n* = 4). ^*^0.01 < *P* < 0.05; ^**^0.001 < *P* < 0.01; ^***^P < 0.001. EVs, Extracellular vesicles.

To define whether serum EVs are cardioprotective *in vivo*, we firstly performed myocardial injection of EVs from mouse serum and ligated the left anterior descending coronary artery in an *in vivo* mouse AMI model. Triphenyl tetrazolium chloride (TTC) staining showed that the area at risk (AAR) relative to left ventricular (LV) was the same under PBS or EV treatment, while the infarct size (INF)/AAR ratio was reduced significantly by EVs (Figures 6A–C). Then, to confirm whether serum EVs could change the expression of miR-21 and its target gene PDCD4 *in vivo*, we firstly detected the expression of miR-21 and PDCD4 in the heart tissues at 24 h after the myocardial injection of EVs from mouse serum. The results showed that miR-21 was significantly elevated while PDCD4 was decreased by mouse serum EVs (Figures 6D–F). When mice were treated with serum EVs, the decrease of miR-21 expression was attenuated and the increase of PDCD4, Bax/Bcl-2 and cleaved Caspase-3/Caspase-3 ratio after AMI was inhibited (Figures 6G–O). These results indicated that serum EVs have crucial roles in increasing miR-21 levels and reducing the infarct size, cell apoptosis in ischemic cardiac tissues during AMI.

## Discussion

Accumulating evidences have suggested that the exogenous EVs from *in vitro* cultured-pluripotent cells are cardioprotective vesicles. Endogenous EVs from serum are protective for myocardium under ischemia-reperfusion injury (Vicencio et al., 2015). Now, our present study, for the first time, suggested that endogenous EVs from serum attenuates cardiomyocytes apoptosis both in OGD/R-stimulated NRCMs and *in vivo* mice models of AMI, regulated by miR-21 that inhibited PDCD4 expression. miR-21 protected H9C2 cells against doxorubicin-induced apoptosis (Tong et al., 2015). Antagonizing miR-21 inhibits interstitial fibrosis and attenuates cardiac dysfunction in a mouse model of heart failure (Thum et al., 2008). In rat AMI model, miR-21 expression was reduced in the infarcted areas after AMI compared with that in other areas and in sham-operated hearts, and miR-21 protect myocardium against AMI by reducing cardiomyocytes apoptosis (Dong et al., 2009). Subsequent study defined that miR-21 was not only cardioprotective for early phase of MI, but also reduced the myocardial fibrosis at 2 weeks after MI in murine model (Gu et al., 2015). To date, miR-21 has been defined as an important regulator of cardiomyocytes apoptosis, cardiac fibrosis and ischemia cardiac diseases. In addition, circulating miR-21 has been identified as a potential biomarker for the diagnosis of AMI (Wang et al., 2017b). Thus, more and more attention has been paid to miR-21 as a therapeutic target for AMI. Given that EVs are the major miRNA carriers in mammalian serum (Zhao et al., 2016), more attention have been paid to the therapeutic effects of EV- encapsulated miR-21 on AMI. Cellular delivery of miR-21 was involved in the improvement of cardiac function after AMI induced by the injection of EVs from MSCs, while miR-21 inhibition in MSCs will cause the absence of therapeutic enhancement of cardioprotective effects by MSC-derived EVs (Wang et al., 2017a). EVs extracted from the culture medium of cardiac progenitor cells protect against cardiomyocytes apoptosis (Xiao et al., 2016). However, EVs used in these two reports are extracted from the *in vitro* systems. Our present study provided the first evidence that EVs from serum have protective effects on myocardium during AMI through miR-21.

The functional significance of miRNAs largely depends on its target genes. In this study, our results indicate that the inhibition of PDCD4 contributes to the anti-apoptotic effects of miR-21 and serum EVs in cardiomyocytes. PDCD4 was first cloned from a mouse cDNA library and was increased during apoptosis in all tested cells lines (Shibahara et al., 1995). Lower levels of PDCD4 were found in normal heart tissues (Onishi et al., 1998). Subsequent research demonstrated that PDCD4 was up-regulated in H2O2-treated cardiomyocytes (Xiao et al., 2016) and served as a pro-apoptotic factor in regulating myocardial apoptosis (Cheng et al., 2010; Jia et al., 2016; Li et al., 2016). In the current study, the expression of PDCD4 in NRCMs is able to be inhibited by miR-21 mimics and serum EVs. In addition, we found that the promotive effects of miR-21 inhibitors on OGD/R-induced cardiomyocytes apoptosis were suppressed by PDCD4 siRNAs. These results were consistent with the previous studies which validated PDCD4 as a direct target gene of miR-21 in cardiomyocytes by luciferase assay (Cheng et al., 2009, 2010). Previous study reported that cardiac progenitor cell-delivered EVs directly targeting PDCD4 in H9C2 cells (Xiao et al., 2016). In this study, we found that co-transfection of PDCD4 siRNAs and human serum EVs has no additive effect in reducing cardiomyocytes apoptosis. Thus, our results indicate that serum EVs also protects against cardiomyocytes apoptosis via the miR-21/PDCD4 axis.

It should be highlighted that this study has several limitations. First, PDCD4 siRNAs could only partially rescue the pro-apoptotic effects of miR-21 inhibitors, indicating other important target genes also existed. Considering one miRNA could directly regulate multiple targets, it would be of great interest to further clarify the other direct targets of miR-21 via bioinformatics prediction and luciferase reports assay in cardiomyocytes. Second, although we have shown serum EVs reduced cardiomyocytes apoptosis through the miR-21/PDCD4 axis *in vitro*, how serum EVs exerted the protective roles *in vivo* still remains to be clarified. Third, miR-21 levels were elevated after treatment with serum EVs. However, whether miR-21 elevation was due to the delivery of miR-21 by EVs or the increased intrinsic expression of miR-21 in cardiomyocytes was still unclear. It will be of great interest to treat miR-21-deficiency cardiomyocytes under OGDR and/or miR-21 knockout mice under AMI with serum EVs, and then detect whether the protective effects of serum EVs were also existed.

In conclusion, we provide novel evidence supporting a critical function of serum EVs in protecting against AMI through miR-21/PDCD4 axis. Our work therefore provides potentially important implications of serum EVs for therapy of AMI.

## Author contributions

HG, ZL, and YL: undertook the western and OGD/R experiments; YX, JY, YZ, and JX: contribute to the exosome purification; HZ and SD: contribute to serum sample collections; QD and CZ: provided technical support in animal experiments. LZ: designed the experiments and contributed to the writing of the paper.

### Conflict of interest statement

The authors declare that the research was conducted in the absence of any commercial or financial relationships that could be construed as a potential conflict of interest.
